# Editorial: Neural and Mechanical Mechanisms in Pulmonary Defense: What Does the Future Hold?

**DOI:** 10.3389/fphys.2022.946768

**Published:** 2022-06-16

**Authors:** Nicolle J. Domnik, John T. Fisher, M. Diane Lougheed, Stuart B. Mazzone, Alice E. McGovern

**Affiliations:** ^1^ Department of Biomedical and Molecular Sciences, Queen’s University, Kingston, ON, Canada; ^2^ Department of Public Health Sciences, Queen’s University, Kingston, ON, Canada; ^3^ Department of Medicine, Queen’s University, Kingston, ON, Canada; ^4^ Department of Anatomy and Physiology, School of Biomedical Sciences, The University of Melbourne, Parkville, VIC, Australia

**Keywords:** cough hypersensitivity, laryngeal dysfunction, chronic cough, cough mechanisms, vagus nerve

## Introduction

Normal respiratory functioning is essential for survival. Injuries or obstructions to airways, lungs or respiratory neural networks can have catastrophic consequences. Every breath places the lungs at the mercy of environmental air quality, which can contain pollutants, noxious gasses, and pathogens, driving or exacerbating acute and chronic respiratory conditions. Airway inflammatory diseases can compromise airflow through excessive mucus and airway wall edema, remodeling, and/or bronchoconstriction. Glottic proximity to the esophagus makes aspiration (during swallowing or from gastric reflux) a constant threat. Indeed, aspiration frequently causes pneumonia and can lead to sudden, lethal airway obstructions. Neurological trauma or pathology may interfere with respiratory processes, with dire consequences for breathing and airway clearance. While impaired sensory function is problematic, so too is hypersensitivity, contributing to conditions such as chronic cough.

It is therefore unsurprising that the respiratory system is equipped with many precise defensive mechanisms that collectively safeguard against risks, but our understanding of these processes is incomplete. This research topic brings together established experts and emerging future leaders to describe the state-of-the-art understanding of pulmonary defenses in health and disease, paving a direction for future research.

## The Research Topic Articles

The respiratory system contains diverse sensory nerve fibers that monitor the physical and chemical environment of the airways and lungs. Subsets of mechanosensitive fibers, the slowly (SARs) and rapidly (RARs) adapting receptors, respond to changes in airway smooth muscle tone and distension and lung volume, providing essential volume-related feedback to brainstem neural networks governing breathing and autonomic neural control. Lin et al. performed single unit RAR and SAR recordings in anesthetized rats, observing that serotonin administration profoundly altered RAR firing, although the response was often delayed and hypothesized to occur secondary to serotonin-induced changes in pulmonary mechanics (Lin et al.). Interestingly, select RARs responded immediately, an effect mediated by 5-HT3 receptors, known to be expressed by some vagal sensory neurons (Hoyer et al., 1989; Rosenberg et al., 1997; Kupari et al., 2019; Mazzone et al., 2020). Serotonin had a lesser effect on SARs; however, when responsive, different SARs subsets responded in a paradoxical fashion. Many RAR and SAR studies have been performed in larger laboratory species. Therefore, the potential of murine models enabling advanced molecular physiology approaches has not yet been fully realized. Accordingly (Domnik et al.) investigated mouse SARs, addressing two fundamental physiological questions. Firstly, mice breathe at significantly higher frequencies than classically employed larger species, suggesting that SAR activity and mechanosensitivity may reflect this. Indeed, murine SARs had lower thresholds of activation, higher discharge frequencies and higher mechanosensitivity compared to larger species. Secondly, it has been proposed that SARs may have terminal fields which directly interact with specialized cells comprising airway neuroepithelial bodies (NEBs). However, Domnik et al. provide strong evidence against this hypothesis in the mouse (Domnik et al.).

Airway sensory nerve plasticity may induce further respiratory pathology and morbidity. The state-of-the-art review by Drake and colleagues summarizes evidence that such plasticity occurs in asthma and chronic cough. Biopsy samples from both patient groups show increased nerve fiber density and changes in neurochemical phenotype, while advances in RNA sequencing, 3D microscopy and physiological assays are beginning to shed light on possible mechanisms involved and eventual functional implications (Drake et al.). Mazzone et al. presented new insights into the potential role of neuroimmune mechanisms in sensory nerve plasticity: a mouse model of severe respiratory viral infection showed vagal sensory neuron mobilization of the alarmin HMGB1 (High Mobility Group Box protein 1) and provided evidence that HMGB1 can act on vagal sensory neurons via its cognate receptor RAGE (receptor for advanced glycation end-products) to induce neuronal firing and increased sprouting of neurites (Mazzone et al.). This provides a potential mechanism for altered sensory neuron sensitivity and structure in pulmonary diseases and may suggest a previously unrecognized target for anti-HMGB1 therapy, currently being developed for use in patients.

Coughing is a critical defensive mechanism for keeping the airways clear from potentially dangerous inhaled, aspirated or locally produced substances. However, altered cough sensitivity leading to excessive coughing is a common, often-debilitating problem. Conversely, insufficient coughing predisposes to early death from aspiration pneumonia. Olsen et al. investigated the cough-suppressing actions of two centrally antitussive drugs (GABA agonist baclofen and opiate codeine) in cats. They showed that neither appears to suppress spinal circuits involved in chest wall and abdominal motor drive, but likely exert their antitussive effects via the brainstem (Olsen et al.). Sood et al. investigated deep breath-induced bronchodilation in patients with chronic cough versus patients with classic asthma, cough-variant asthma and healthy controls. Their surprising finding was that the deep inspiration index was significantly lower in cough with normal airway sensitivity versus individuals with either asthma or healthy controls, suggesting that the bronchodilating effect of deep inspiration is impaired in chronic cough without asthma (Sood et al.). Bai et al. provided an overview of the perplexing problem of why women are more likely to develop chronic cough than men and disproportionately prone to poor quality of life resultant from cough. Evidence for mechanistic and sociological differences is presented, providing a strong interrogation of sex- and gender-differences in a common respiratory condition and highlighting the need for more research into this important clinical observation (Bai et al.). Sykes and Morice reviewed the state of play in chronic cough and cough hypersensitivity clinical research, including the current pipeline of antitussives. They contrasted this with the topic of cough hyposensitivity, highlighting the ‘two faces’ of cough presented to clinicians and the challenge of developing effective antitussives that suppress unwanted coughing while preserving defensive cough needed to protect against aspiration (Sykes and Morice).

## Conclusion

Defense of the pulmonary system is inarguably essential; however, more research is required to fully elucidate the mechanisms and consequences of pulmonary defensive processes. In submissions originating from four continents and reflecting the efforts of established experts and emerging researchers, this research topic describes the cutting-edge understanding of pulmonary defenses in health and disease. Readers are provided with insight into processes including cough hyper and hypo -sensitivity, sensory neural mechanisms, and sex differences in pulmonary defense, providing strong foundations for future inquiry.
